# Irrigation Regime Effects on Phenolic Composition of Portuguese Grape Varieties

**DOI:** 10.3390/molecules30163408

**Published:** 2025-08-18

**Authors:** Daniela Fonseca, Rosario Sánchez-Gómez, M. Rosario Salinas, Maria João Cabrita, Nuno Martins, Raquel Garcia, Cristina Cebrián-Tarancón

**Affiliations:** 1 Mediterranean Institute for Agriculture, Environment and Development & Institute of Research and Advanced Training, University of Évora, Pólo da Mitra, Ap. 94, 7006-554 Évora, Portugal; daniela.fonseca@uevora.pt; 2 Cátedra de Química Agrícola, E.T.S. Ingeniería Agronómica y de Montes y Biotecnología, Departamento de Ciencia y Tecnología Agroforestal y Genética, Universidad de Castilla-La Mancha, Avda. de España s/n, 02071 Albacete, Spain; rosario.sgomez@uclm.es (R.S.-G.); rosario.salinas@uclm.es (M.R.S.); 3 Mediterranean Institute for Agriculture, Environment and Development & Global Change and Sustainability Institute, University of Évora, Pólo da Mitra, Ap. 94, 7006-554 Évora, Portugal; mjbc@uevora.pt (M.J.C.); nmartins@uevora.pt (N.M.); raquelg@uevora.pt (R.G.); 4 Department of Crop Science, School of Science and Technology, University of Évora, Pólo da Mitra, Ap. 94, 7006-554 Évora, Portugal

**Keywords:** autochthones varieties, grape quality, HPLC-DAD, water supply, phenolic compounds, sustainable viticulture

## Abstract

Climate change has led to increased extreme weather events, such as severe droughts and intense rainfall, with regions in Portugal, like Alentejo and Algarve, being particularly affected. Understanding the influence of water availability in the concentration of phenolic compounds in autochthonous varieties could be an important tool to know how these varieties adapt to water scarcity. This work has been carried out with the aim to analyze the profile of phenolic compounds by HPLC-DAD in four Portuguese grape varieties (Tinta Gorda, Tinta Miúda, Tinta Caiada, and Moreto), cultivated under three irrigation regimes (water comfort, moderate water deficit, and rainfed). The results reveal that Tinta Gorda, Tinta Miúda, and Tinta Caiada varieties exhibit the higher concentrations of phenolic compounds under rainfed conditions. Among these, Tinta Miúda and Tinta Caiada stand out as the most promising varieties in terms of adaptability to water scarcity.

## 1. Introduction

In recent years, there have been extreme climatic phenomena in various regions of the world, such as periods of extreme drought or periods when it rains heavily in a short period. These phenomena have become increasingly frequent because of climate change, and Portugal is not an exception. The Alentejo and Algarve regions have been increasingly affected by periods of extreme drought, and so adapting to the lack of water is becoming more essential [1,2,3,4,5]. Some winegrowers have already begun to reduce their vineyard irrigation due to limitations in the amount of water available for irrigation. Other producers have even opted to give up irrigation, leaving the vineyards totally dependent on rainfall. However, these changes in the amount of irrigation to be applied to the vines can have consequences, such as early ripening or low production yields, affecting the quality of the grapes and, consequently, the wines [4,6]. These consequences are less noticeable when the vines are produced from autochthonous varieties, which are usually better adapted to extreme conditions, such as lack of water. In fact, these varieties can adapt to these conditions because, when they are under water stress, they normally close their stomata to reduce water loss through evapotranspiration from the leaves, causing the vines to synthesize more phenolic compounds [7,8,9].

Phenolic compounds are essential for the quality of grapes and wines and are classified into two main groups—flavonoids and non-flavonoids. Flavonoids include flavonols, anthocyanins, flavan-3-ols (also known as flavanols), flavanones, flavones, and isoflavones, while non-flavonoids comprise stilbenes and hydroxycinnamic acids [10,11]. Anthocyanins play a crucial role in red grapes and wines, as their concentration directly influences wine color. Flavan-3-ols contribute to the wine’s structure, astringency, and bitterness, while flavonols primarily enhance color [12,13,14]. Phenolic compounds are the best-known secondary metabolites in grapes, as they are characterized by a wide range of unique bioactive properties, including antioxidant, anti-inflammatory, anticarcinogenic, antiproliferative, and antibacterial properties [15,16,17,18].

Understanding how climate change affects phenolic compounds is of great importance [19]. According to the study realized by Tarara et al., 2008 [20] on Merlot grapes, higher berry temperatures led to an increased concentration of malvidin-based anthocyanins, driven primarily by the accumulation of malvidin-3-coumaroyl-glucoside. However, other studies have reported that temperature did not influence the accumulation of malvidin-3-glucoside in Pinot Noir or Merlot grapes [21,22]. The literature also indicates that moderate water deficit improves grape quality attributes and, consequently, wine composition [23,24]. According to Alatzas et al., 2021 [9] the anthocyanins and phenols in berries increased during ripening and were higher in non-irrigated plants compared to irrigated ones in the Agiorgitiko variety. Esteban et al., 2001 [25] reported that grapevines grown without irrigation tended to accumulate greater amounts of total phenols and tannins in their skins compared to those subjected to irrigation. Furthermore, exposing grapevines to water deficit conditions results in elevated levels of anthocyanins and phenols [26,27]. According to Ramos et al., 2024 [6] water availability influenced the concentration of anthocyanins but had no significant impact on flavonol levels. According to Pérez-Álvarez et al., 2021 [8] irrigation regimes had varying effects on the content of phenolic compounds. In a study on Monastrell plants subjected to different irrigation conditions, the highest concentrations of anthocyanins, flavonols, flavanols, hydroxybenzoic acids, and total phenolic compounds were observed under dry conditions. In contrast, the total concentration of hydroxycinnamic acids, stilbenes, and total non-flavonoids were not affected. The high phenolic compound content of grapes makes them an important source of natural antioxidants, among other beneficial health properties. Vineyard irrigation might affect berry composition and quality. Regulated deficit irrigation (RDI) is a widely used strategy to reduce the possible negative impact of irrigation on grapes, improving grape composition and resulting in water savings. Monastrell grapevines (*Vitis vinifera* L.) grown in eastern Spain were subjected to two water regime strategies—rainfed (non-irrigation) and RDI. The content of anthocyanins, flavonols, flavanols, hydroxybenzoic and hydroxycinnamic acids, and stilbenes was determined by HPLC and was related with total phenolic content and three antioxidant activity methods (ABTS, DPPH, and ORAC). The study aimed to evaluate and compare the phenolic composition and antioxidant potential of Monastrell grapes. The rainfed regime concentrated grapes in terms of phenolic compounds. Thus, the total content of anthocyanins, flavonols, flavanols, hydroxybenzoic acids, and total phenols were higher in the rainfed grapes than in the RDI ones. Additionally, the rainfed grapes doubled their antioxidant potential with respect to the RDI grapes with the ORAC method. Total phenolic content and antioxidant activity by ORAC assay positively correlated with most of the total phenolic compounds analyzed. This study demonstrates how field practices can modulate final grape composition in relation to their antioxidant activity [8]. Rouxinol et al., 2023 [7] reported that lower rainfall and higher temperatures affected berry size and phenolic composition by increasing polyphenol concentration. These studies highlight the diversity of results obtained, which vary according to irrigation conditions, the varieties, and the climate.

One of the alternatives for dealing with these climate change scenarios is the study and recovery of autochthonous varieties, since they possess specific characteristics and adaptations to their development environment. So, understanding the impacts of climate change on the grape quality from autochthonous varieties is key to finding a balance between the need for climate resilience, safeguarding water resources, and the desire to preserve wine quality and authenticity [28]. This line of enquiry, that autochthonous varieties are better adapted to conditions of water and heat stress and are more resilient to heat waves, is widely studied [29]. In Portugal, among these varieties are Tinta Caiada, Tinta Gorda, Tinta Miúda, and Moreto, all of them red varieties. The adaptability of these varieties to hot and dry climates makes them particularly relevant in the context of climate change. However, to our knowledge, the study of their phenolic content under different water regimes has not yet been carried out. Tinta Caiada and Tinta Gorda are characterized by their sensitivity to rot and prefer warm and dry climates for optimum development. Moreto is a vigorous grape and productive variety, adapting well to hot and dry climates, grown mainly in the Alentejo [30,31,32]. The Tinta Miúda variety is known for producing wines with an intense color and rich tannins [33,34,35,36].

For this reason, the aim of this work was to analyze the profile of phenolic compounds in four Portuguese red grape varieties, Tinta Gorda, Tinta Miúda, Tinta Caiada, and Moreto, cultivated under three irrigation regimes (water comfort, moderate water deficit, and rainfed) by high-performance liquid chromatography coupled to a diode array detector (HPLC-DAD).

## 2. Results and Discussion

### 2.1. Internal Validation of the HPLC-DAD Method

The results obtained in the validation method are shown in Table 1. The linearity assessment of the proposed method was carried out through calibration curves of the standards used, which were constructed with five points, and their linearity was determined using the least squares regression method (R^2^). All calibration curves exhibited correlation coefficients above 0.99, except for quercetin, which had an R^2^ value of 0.98, demonstrating excellent overall linearity. According to Padilha et al., 2017 [37] the R^2^ values for calibration curves must exceed 0.99, confirming the linearity achieved in this study. The method’s sensitivity was evaluated based on the limits of detection (LOD) and quantification (LOQ) values (Table 1), which ranged from 0.07 to 11.95 mg L^−1^ for LOD and from 0.17 to 31.61 mg L^−1^ for LOQ, confirming its capability to accurately quantify each phenolic compound. Regarding repeatability, all relative standard deviation (RSD) values were below 5.54%, while for reproducibility, the RSD values were below 7.91%, demonstrating that the method was precise. Gallic acid showed the highest values in both cases, with 5.54% and 7.91% for repeatability and reproducibility, respectively, whereas viniferin showed the lowest repeatability value at 0.46%, and caffeic acid had the lowest reproducibility value at 0.29%. According to Moratalla-López et al., 2020 [38] RSD values of less than 8% represent very good precision, being all the RSD obtained in the validation below of this value. Regarding accuracy, the average recovery of malvidin-3-glucoside at three different concentrations (75%, 85%, and 100%) was 80.39%. These findings indicate the satisfactory accuracy of the proposed method. Therefore, considering the performance characteristics assessed, including linearity, selectivity, LOD, LOQ, precision, and accuracy, the HPLC-DAD method was successfully validated.

### 2.2. Oenological Results of Samples

Table 2 presents the oenological parameters of the grape varieties on the day of harvest according to the different irrigation regimes studied. The Tinta Gorda variety exhibited similar potential alcohol degree and pH values across all irrigation regimes. However, total acidity was higher in the water comfort regime, while similar values were observed for the moderate water deficit and rainfed regimes. Tinta Miúda stands out with the highest potential alcohol degree and total acidity among the varieties studied, with both parameters increasing as water availability decreases. Conversely, pH values remain consistent across irrigation regimes. According to the limited literature available on the oenological parameters of the studied grape varieties, the total acidity of Tinta Miúda musts is generally high, ranging on average from 5.5 to 7.5 g L^−1^. The potential alcohol content typically varies between 12 and 13% *v*/*v* [30]. For the Tinta Caiada variety, potential alcohol degree and pH increased as water availability decreased, while total acidity showed the opposite trend. According to Böhm et al. [30], total acidity averages between 3.5 and 5.5 g L^−1^, while the potential alcohol content ranges from 11.5 to 14% *v*/*v*. These findings are consistent with the results obtained in the present study for this variety. The Moreto variety exhibited the lowest potential alcohol degree among the studied varieties, with only slight variations in other parameters across the different irrigation regimes. For this variety, the literature reports that both the potential alcohol degree and total acidity are generally low, with average values around 10.5% *v*/*v* and 3.5 to 4.8 g L^−1^, respectively. Unfortunately, it seems that the degree of maturation was slightly lower from what is expected according to the literature [30].

Since grapes were harvested in the same day and have grown under the same edapho-climatic conditions and the same agronomic practices, the results in Table 1 show the different maturation behavior of these varieties.

### 2.3. Low-Molecular-Weight Phenolic Compounds in Grapes

A total of 19 phenolic compounds including 9 anthocyanins, 6 flavanols, 2 flavonols, and 2 other phenolic compounds were identified and quantified across the varieties Tinta Gorda, Tinta Miúda, Tinta Caiada, and Moreto under different irrigation regimes.

**Figure 1:**
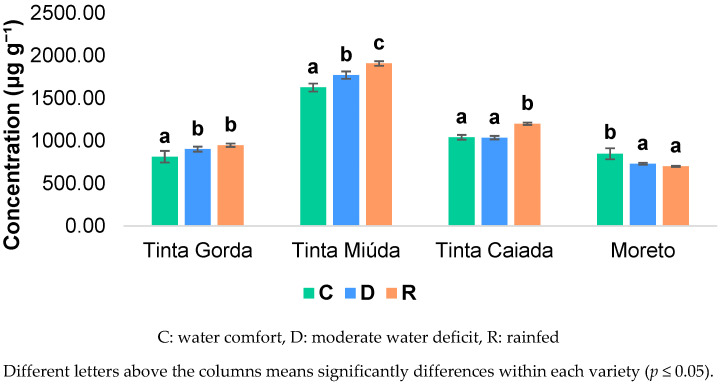
Total concentration (µg g^−1^) of low-molecular-weight phenolic compounds according to irrigation regime for each variety.

Figure 1 presents the total phenolic compounds of the four studied varieties, with Tinta Miúda showing the highest concentrations, regardless of the water regime. This variety was the only one that shows statistically significant differences in terms of the total concentration of phenolic compounds between all the irrigation regimes, with the concentration increasing as the amount of water available from irrigation decreases. Thus, as the total concentration of phenolic compounds increases as water availability decreases, it can be deduced that the Tinta Miúda variety shows a good adaptability in conditions of water deficit or even rainfed. On the other hand, Tinta Gorda showed no differences between the moderate water deficit and rainfed irrigation regimes, but these differ significantly from the water comfort irrigation regime. Tinta Gorda’s enhanced phenolic compound concentrations under moderate water deficit and rainfed conditions suggest that this variety is well adapted to water scarcity. The Tinta Caiada variety, on the other hand, was the only one who did not show significant differences between the regimes of water comfort and moderate water deficit. However, both regimes were statistically different from the rainfed irrigation regime. Furthermore, it is for the rainfed irrigation regime that this variety showed the highest concentration of phenolic compounds, suggesting good adaptability to conditions of water scarcity. For Moreto the concentration of phenolic compounds decreased when the water supply was lower. Therefore, the lower concentrations of phenolic compounds observed in this variety under moderate water deficit and rainfed irrigation suggest that, among all the varieties analyzed, Moreto has the highest difficulty adapting to water scarcity, perhaps due to its low degree of maturity.

Table 3 presents the average concentrations of each compound, along with the results of the ANOVA analysis, which evaluates the differences between the irrigation regimes for each variety, for the year 2024. In general, the effect of the irrigation regime on the phenolic composition of Portuguese grape varieties is notable for the varieties under study. Anthocyanin content was higher in the Tinta Miuda variety, regardless of the irrigation regime, while the lowest levels were observed in the Moreto variety. These results are related to the degree of ripeness of the grapes. The pattern was similar for Tinta Miuda and Tinta Caiada, with the rainfed grapes showing higher concentrations. On the other hand, Tinta Gorda and Moreto exhibited distinct anthocyanin profiles. In Tinta Gorda, concentrations were lower under water comfort conditions, with no significant differences observed between moderate water deficit and rainfed conditions. Conversely, the Moreto variety displayed a pattern opposite to that of the other cultivars, with the highest levels recorded under water comfort conditions, suggesting a lower efficiency in anthocyanin synthesis in response to water stress. However, such differences between varieties could also be due to genetic characteristics. Individually, the compound with the highest concentration was malvidin-3-glucoside, and its behavior with respect to the water supply followed the same trend as that of the total anthocyanins. Costa et al., 2014 [13] analyzed the anthocyanin composition of 24 grape varieties grown in Portugal and found that malvidin-3-glucoside and malvidin-3-coumaroyl-glucoside were the predominant anthocyanin for most varieties. Ollé et al., 2011 [39] revealed that despite the different irrigation treatments, malvidin-3-glucoside and its coumarate derivative were the most abundant compounds in Syrah grapes. Such findings suggest that climate plays a role in shaping the phenolic compounds profile, as water stress conditions stimulated anthocyanin biosynthesis [5,27,40].

The flavanol content was higher in the Tinta Miúda variety, regardless of the irrigation regime. The pattern was similar for Tinta Miúda and Tinta Gorda, with rainfed grapes showing higher concentrations. As for the other varieties, Moreto had a higher flavanol content under conditions of moderate water stress, while Tinta Caiada had a higher flavanol content under the water comfort regime. Among the individual compounds, epicatechin gallate and procyanidin 2 showed the highest concentrations in all the varieties studied. According to Pérez-Álvarez et al., 2021 [8] for Monastrell grapes, only the concentrations of epicatechin and epicatechin-3-gallate increased significantly under the rainfed treatment compared to regulated deficit irrigation. According to Theocharis et al., 2021 [41] irrigation influenced the amount of flavanols in berries, with the highest levels observed in non-irrigated vines and those where irrigation began at mid-ripening. In contrast, the lowest levels were found in vines irrigated immediately after veraison and in those subjected to continuous irrigation from berry set to harvest. Also, it was possible to semi-identify one flavanol and two procyanidins, whose behavior was similar to the rest of the flavanols previously indicated.

Regarding to flavonols, two compounds were detected, but they could not be identified. Their total content was higher in the Tinta Miúda variety, independent of the irrigation regime, whereas the Moreto variety consistently showed the lowest levels, which is to be expected, as it is the variety with the lowest degree of ripeness. However, genetic differences between varieties may also be related to these results. Tinta Caiada exhibited greater concentrations under rainfed conditions, while Tinta Miúda and Moreto had higher flavonol levels under moderate water comfort. In contrast, Tinta Gorda displayed a distinct pattern, reaching peak concentrations under full water comfort conditions. Ramos et al. [6] observed that in Merlot grapes, water availability does not have a significant impact on flavonol content. Besides, Zarrouk et al. [42] revealed that the Aragonês variety had higher flavonol levels when water availability was greater. This may suggest that the autochthonous varieties under study are better adapted to conditions of water scarcity than traditional ones.

Caffeic acid and resveratrol piceid were included in the “other compounds” group. For caffeic acid, its total concentration was higher in the Tinta Miúda variety, regardless of the irrigation regime. The pattern was similar for Tinta Gorda and Tinta Caiada, with grapes from water deficit and rainfed regimes showing higher concentrations, while for Moreto, there were differences between all irrigation regimes, with water comfort showing the highest concentration for such compounds. The Tinta Miúda variety showed the most distinctive behavior, since it was possible to identify a stilbene, resveratrol piceid, only in this variety. The literature mentions that the total concentration of stilbenes, hydroxycinnamic acids, and hydroxybenzoic acids did not show significant differences between the non-irrigated and regulated deficit irrigation regimes, except one compound, *trans-*fertaric acid [8]. This highlights the value of the autochthonous varieties under study, as although only one hydroxycinnamic acid (caffeic acid), it was found across all varieties, showing significant differences between the irrigation regimes in every case. Furthermore, the stilbene, resveratrol piceid, identified and quantified exclusively in the Tinta Miúda variety, provided insights into the potentially distinctive antioxidant capacity of this variety compared to the others analyzed [34,36].

Considering all the above, it should be noted that the Tinta Miúda grape variety could be better adapted to rainfed conditions. This variety also stands out for its exceptionally high anthocyanin content, which is almost double the combined concentration of the other families of compounds, consistent with the literature describing its tendency to produce deeply colored wines. In fact, such variety is often used in combination with others in the production of high-quality red wines. This color stability may be attributed, in part, to its elevated levels of catechins and proanthocyanidins. Furthermore, Tinta Miúda stands apart from other varieties due to the exclusive presence of resveratrol piceid, suggesting that wines produced from this grape may possess enhanced antioxidant capacity [33,34,35,36]. Wines produced from Tinta Miúda grapes are generally of high quality, with intense color and robust structure. They exhibit an initial astringency that gradually softens with aging, indicating a good aging potential and resulting in wines that become increasingly pleasant and balanced over time [43]. These sensory attributes are closely linked to the phenolic composition of the wines, particularly tannins and anthocyanins, which play key roles in astringency, bitterness, and color stability. Variations in phenolic content, therefore, significantly influence the sensory expression and aging behavior of different grape varieties. Moreto is a traditional grape variety from the Alentejo region, known for its low phenolic content and the low color intensity of the wines it produces. These characteristics were confirmed by Cabrita [44], who reported low anthocyanin concentrations in wines made from this variety, which may result in reduced astringency but also limited color stability and aging potential. Similarly, Tinta Gorda produces wines with low color intensity, along with red fruit notes, a delicate and persistent profile, and a limited capacity for aging [45]. This can be attributed to its lower levels of phenolic compounds, especially anthocyanins and condensed tannins, which are essential for the structural backbone and color retention of red wines during bottle aging. In contrast, Tinta Caiada wines, when harvested at optimal ripeness, express intense, refined, and well-balanced aromas, including notes of plum, raisins, and wild berries. These wines are smooth and delicate on the palate, with a harmonious finish. While they exhibit a light garnet color, their sensory quality can be enhanced if phenolic development, particularly in terms of flavanols and anthocyanins, is optimized through viticultural and enological practices [46].

### 2.4. Principal Component Analysis

Principal component analysis (PCA) has been employed to reduce multiple independent variables into principal component variables with eigenvalues greater than 1 [47,48,49]. In all of them, two main components were selected to visualize distribution of the samples in the factor plane, and the variables with the highest weights are listed in Table 4 for each water regimen considered.

**Figure 2:**
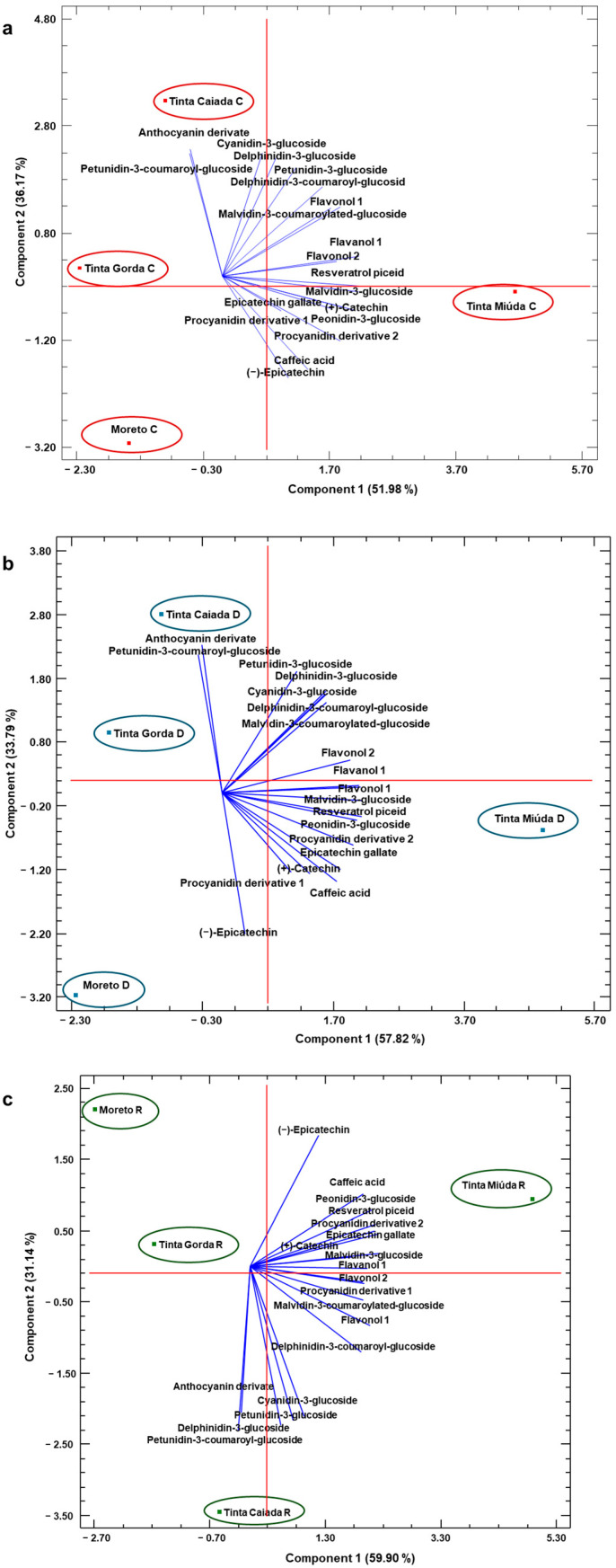
Principal component analysis (PCA) of grapes varieties using the low-molecular-weight phenolic compounds concentrations. The projection of the grape samples on the plane formed by the two principal components is shown. (**a**) water comfort (C); (**b**) moderate water deficit (D); and (**c**) rainfed (R).

In Figure 2a, related to water comfort, components 1 (PC1) and 2 (PC2) explain 88.15% of the total variability, accounting for 51.98% for component 1 and 36.17% for component 2. There was a clear differentiation between the Tinta Miúda variety and the other varieties under study according to component 1, while according to component 2 there was a differentiation between Tinta Caiada and Tinta Gorda in the positive part and Tinta Miúda and Moreto in the negative one. PC1 was primarily characterized by positive loadings of resveratrol piceid and flavanol 1 (Table 4). In contrast, the variables that most strongly influenced the differentiation along PC2 were anthocyanin derivate and cyanidin-3-glucoside. As for the negative charges, the variables that most strongly influenced differentiation were epicatechin and caffeic acid, along with PC2. In Figure 2b, components 1 and 2 explain 91.61% of the total variability, accounting for 57.82% for component 1 and 33.79% for component 2. In this case, the differentiation in two components was similar to that obtained in comfort regime. So, the moderate water deficit regime showed a clear differentiation between Tinta Miúda and the other varieties, according to component 1. The compounds that contributed most positively to differentiation along PC1 were resveratrol piceid and malvidin-3-glucoside, whereas for PC2, the main contributing compounds were petunidin-3-coumaroyl-glucoside and anthocyanin derivate (Table 4). Regarding the negative charges, the variables that contributed most significantly to the differentiation along PC2 were caffeic acid and (–)-epicatechin. On this part, in Figure 2c related to rainfed regime, components 1 and 2 capture 91.04% of the total variability, accounting for 59.90% for component 1 and 31.14% for component 2. According to component 2, there was also differentiation, but this time between the Moreto, Tinta Miúda, and Tinta Gorda varieties in the positive part and Tinta Caiada in the negative one. PC1 was defined by positive contributions from epicatechin gallate and malvidin-3-glucoside. Conversely, the variables that contributed most to the differentiation along with PC2 were (–)-epicatechin and caffeic acid. Concerning the negative charges, cyanidin-3-glucoside and petunidin-3-coumaroyl-glucoside were the variables that had the greatest impact on differentiation along PC2 (Table 4).

## 3. Materials and Methods

### 3.1. Chemicals

All solvents and reagents used were of HPLC-grade or analytical-grade quality. Ultrapure water was obtained using a Milli-Q system (Millipore, Bedford, MA, USA). Standards used was purchased, as follows: gallic acid, 4-hidroxybenzoic acid, protocatechuic acid, (+)-catechin, vanillic acid, caffeic acid, sinapic acid, syringic acid, coumaric acid, (−)-epicatechin, epigallocatechin gallate, epicatechin gallate, catechin gallate, piceatannol, procyanidin, viniferin, malvidin-3-glucoside, and resveratrol piceid from Sigma-Aldrich (Steinheim, Germany) and ferulic acid, trans-resveratrol, and quercetin from TCI (Tokyo, Japan). Formic acid was purchased from Sigma-Aldrich (Steinheim, Germany), acetonitrile from Carlo Erba (Val de Ruil Cedex, France), ethanol from Honeywell (Seelze, Germany), and methanol from PanReac (Darmstadt, Germany).

### 3.2. Sample Material

**Figure 3:**
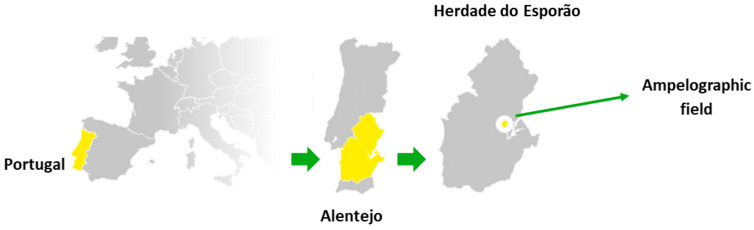
Map of the geographical location of the vineyard where the grapes were harvested.

Tinta Gorda (VIVC: 8082), Tinta Miúda (VIVC: 26725), Tinta Caiada (VIVIC: 8951), and Moreto (VIVC: 7992) grapes from the Esporão ampelographic field at Herdade do Esporão in Reguengos de Monsaraz (Alentejo) were all harvested on 27 August 2024. The map showing the location of the vineyard is presented in Figure 3. The ampelographic field at Esporão is an experimental vineyard; planted in 2011, it is based on granodiorite soils, with a planting density of 2222 plants per hectare in a bilateral Cordon de Royat system, oriented north–south and managed under organic farming, ensuring balance, good sun exposure, and sustainability in grape production. Vineyards were subject to the following three different irrigation regimes: water comfort (C), moderate water deficit (D), and rainfed (R). The water comfort regime applied 2686 m^3^/ha of water, moderate water deficit applied 1343 m^3^/ha, while the rainfed regime relies solely on natural precipitation. A random sample of grapes was collected from each variety under each irrigation regime. That is, for each variety, three samples were taken, one under the water comfort regime, one under the moderate water deficit regime, and one under the rainfed regime. Fresh must was used for measure oenological parameters, and grapes were frozen at −32 °C until further analysis.

### 3.3. Climatic Conditions

**Figure 4:**
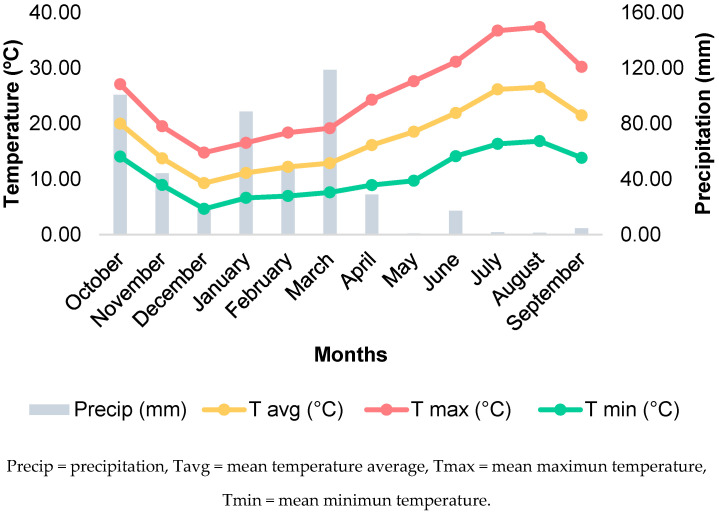
Temperature and precipitation in 2024, in the ampelographic field of Herdade do Esporão.

Due to the impact of climate conditions on vine development and ripening, Figure 4 presents the results of precipitation and temperature throughout the 2024 agricultural year. The months with the highest rainfall were March, October, and January. Starting in May, precipitation dropped to less than 20 mm, which is expected, as these are the summer months. Regarding temperatures, winter months showed lower values, while temperatures increased from spring. Between June and September, maximum temperatures ranged from 30 °C to 37 °C. The data reflect a typical seasonal pattern, with a rainy winter and spring, followed by a hot and dry summer. Total precipitation for the 2024 agricultural year was 474 mm.

### 3.4. Oenological Parameters

Oenological parameters, such as potential alcohol degree (% *v*/*v*), total acidity (g L^−1^ of tartaric acid), and pH, were determined following the guidelines established by the International Organization of Vine and Wine (OIV) [50]. The probable alcohol content was determined using a HI96813 refractometer (Hanna Instruments, Nusfalau, Salaj County, Romania) by simply placing a few drops of must onto the refractometer. For the determination of total acidity and pH, 25 mL of must was transferred to a glass, and measurements were performed using a CRISON Compact Titrator (Crison Instruments S.A., Barcelona, Spain).

### 3.5. Sample Preparation

Grapes were unfrozen and crushed using a blender (Ufesa BS4717 Ruby Red, Barcelona, Spain). An amount of 50 g of previously crushed grapes were weighed, followed by the addition of 25 g of ethanol, and the mixture was stirred for 1 h at 600 rpm in a multiposition magnetic stirrer (Twister^®^, Gerstel, Mülheim, Germany). The samples were then filtered, and the liquid was collected in falcon’s tubes. Next, the liquid was centrifuged at 4000 rpm for 10 min. After centrifugation, the volume of liquid was measured for each sample. Finally, the samples were filtered through a 0.22 μm PVDF filter (LLG Labware, Meckenheim, Germany). For each variety and irrigation regime, three samples were performed.

### 3.6. HPLC-DAD Analysis

To determine the low-molecular-weight phenolic compounds of grapes, the procedure was based on the previous method developed by Cebrián-Tarancón et al., 2019 [51] to which minor modifications were made. Analyses were performed using an Agilent 1200 HPLC chromatograph (Agilent Technologies, Palo Alto, CA, USA) equipped with a Diode Array Detector (DAD, Agilent G1315D, Agilent Technologies) coupled to an Agilent ChemStation (version B.03.01) data processing system. Separation was performed using a reverse-phase TRACER EXCEL PFPEC (4.6 mm × 150 mm, 3 μm particle size) coupled with an ACE Excel HPLC pre-column Filter 1PK (0.5 μm particle size), maintained at 30 °C. The mobile phase consisted of phase A composed of 1% acetonitrile, 1.5% formic acid, and water miliQ, and phase B was prepared with 20% phase A, 1.5% formic acid, and acetonitrile. The following elution gradient was used: 0–8.4 min, 5% B, 8.4–12.5 min, 5–10% B; 12.5–13.5 min, 10%, 13.5–24 min, 10–15% B; 24–30 min, 15% B; 32 min, 16% B; 36 min, 19% B; 45 min, 32% B; 50 min, 90% B; 52 min, 100% B; 54 min, 100% B; 58 min, 5% B; 60 min, 5% B. The flow rate was set at 1 mL/min, with a sample injection volume of 20 µL. The DAD was set at 256, 280, 308, 324, 365, and 520 nm.

A total of 21 phenolic compounds were analyzed in grapes, which were identified based on their UV-Vis spectra and retention times of their corresponding pure standards (Sigma-Aldrich, Steinheim, Germany). Anthocyanins were detected and quantified at 520 nm, flavanols at 280 nm, phenolic acids at 256, 280, and 324 nm, stilbenes at 308 and 324 nm, and flavonols at 365 nm.

### 3.7. Internal Validation of the HPLC-DAD Method

Intra-laboratory validation of the method was conducted following the Eurachem guide (Magnusson and Ornemark, 2014 [52]). The evaluated performance characteristics included linearity, selectivity, limits of detection (LOD) and quantification (LOQ), precision, and accuracy. Calibration curves were performed to evaluate linearity for the 21 phenolic compounds. These curves were generated by injecting standard solutions at five different concentration levels, with each concentration analyzed in triplicate. The LOD and LOQ were calculated at the lowest concentrations as the concentrations that yielded signal-to-noise (S/N) ratios of 3 and 10, respectively.

The precision method was assessed by examining both repeatability and reproducibility. For repeatability, each standard at the intermediate concentration from the calibration curves was injected 6 consecutive times along the same day, with the same instrument and operator. Reproducibility was determined by injecting the intermediate concentration of each standard 10 times over five separate days. The method’s accuracy was evaluated through a recovery test, focusing on the anthocyanin malvidin-3-glucoside, given that it was the major compound. This analysis was conducted on Moreto grape samples subjected to moderate water deficit, with tests performed in triplicate. After extraction, three different amounts of the malvidin-3-glucoside standard (75%, 85%, and 100% of the initial concentration) were used to extracts fortification. Additionally, a Moreto grape under moderate water deficit, without standard addition, was injected as a baseline. The percentage recovery was determined using the formula outlined by Magnusson and Örnemark [52].

### 3.8. Statistical Analysis

NCSS 11 Statistical Software (2016) (LLC, Kaysville, UT, USA) was used to perform a one-way analysis of variance (ANOVA) to evaluate the impact of irrigation regimes water comfort, moderate water deficit, and rainfed on the phenolic composition of the Tinta Gorda, Tinta Miúda, Tinta Caiada, and Moreto varieties. Differences were considered significant at a probability level of 0.05 using the Tukey–Kramer multiple-comparison test. The influence of the irrigation regime on each variety was evaluated by principal component analysis (PCA), which was performed using the Statgraphics Centurion statistical program (version 19.4.02, StatPoint, Inc., The Plains, VA, USA).

## 4. Conclusions

The results of this study indicate that the Tinta Miúda variety exhibited a remarkable capacity to adapt to the edaphoclimatic conditions of the Alentejo region, characterized by high temperatures, intense solar radiation, and limited water availability. Compared to the other varieties analyzed, Tinta Miúda appeared to be the most adapted to moderate water deficit conditions or even rainfed cultivation, which are increasingly common in semi-arid climates. This performance is largely attributed to its high content of phenolic compounds, particularly anthocyanins, flavanols, and stilbenes, which contribute to greater color intensity, antioxidant potential, and overall complexity of the wines. Additionally, Tinta Miúda maintained elevated levels of total acidity even under high-temperature conditions, an essential trait for ensuring sensory balance, microbial and chemical stability, and aging potential. Therefore, Tinta Miúda emerges as a promising variety to produce high-quality wines in regions facing thermal and hydric stress. Nevertheless, it is important to emphasize that these findings are based on data from a single-year trial and should be regarded as preliminary. Long-term and multi-season studies are necessary to confirm these trends and to further elucidate the behavior of this variety under varying environmental conditions.

## Figures and Tables

**Table 1:** Parameters of method validation: regression curve, linearity, limits of detection and quantification, and repeatability and reproducibility of the experimental procedure for HPLC-DAD analysis.

Standards	Detection Wavelength (nm)	Retention Time (min)	Concentration Range (mg L^−1^)	Regression Equation	R^2^	LOD (mg L^−1^)	LOQ (mg L^−1^)	Repeatability (% RSD)	Reproducibility (% RSD)
Gallic acid	280	3.70	3.91–55.90	y = 53.23x − 13.98	0.9997	0.30	0.37	5.54	7.91
Protocatechuic acid	280	6.42	1.41–20.10	y = 32.57x − 3.09	0.9998	0.17	0.34	4.32	1.27
4-Hidroxybenzoic acid	256	10.46	9.71–138.76	y = 109.89x − 18.79	0.9998	0.20	0.28	0.78	0.38
(+)-Catechin	280	14.11	7.54–107.78	y = 10.85x − 8.34	0.9996	1.06	1.75	2.94	0.82
Vanillic acid	256	15.32	7.41–105.92	y = 64.68x − 15.35	0.9998	0.29	0.40	0.76	0.49
Caffeic acid	324	15.83	3.21–45.86	y = 104.84x − 29.13	0.9997	0.34	0.50	0.88	0.29
(−)-Epicatechin	280	18.76	10.19–145.58	y = 12.87x − 3.74	0.9999	0.52	1.04	1.29	0.53
Syringic acid	280	18.65	1.84–26.32	y = 60.63x − 7.94	0.9998	0.19	0.32	0.52	0.50
Coumaric acid	308	22.23	1.05–14.99	y = 151.65x − 13.14	0.9996	0.11	0.17	3.54	0.85
Ferulic acid	324	27.49	14.83–211.90	y = 101.50x − 34.26	0.9999	0.43	0.65	0.65	5.28
Resveratrol piceid	308	29.79	1.94–27.76	y = 88.72x − 17.16	0.9996	0.24	0.35	2.40	0.47
Sinapic acid	324	31.26	5.36–76.56	y = 96.69x − 43.70	0.9999	0.60	0.94	1.53	1.70
*trans*-Resveratrol	308	44.62	1.31–18.67	y = 142.98x − 52.59	0.9982	0.39	0.43	0.69	4.79
Quercetin	365	48.37	0.66–9.39	y = 60.81x − 36.92	0.9808	0.62	0.65	1.20	1.19
Malvidin-3-glucoside	520	28.19	19.86–397.10	y = 48.65x − 171.75	0.9976	11.95	31.61	0.48	1.57
Procyanidin	280	15.75	10.00–160.00	y = 7.96x − 14.35	0.9991	2.16	2.99	0.50	1.12
Epigallocatechin gallate	280	22.92	3.00–48.00	y = 10.81x − 3.82	0.9974	0.79	1.80	1.76	6.65
Epicatechin gallate	280	31.28	3.00–48.00	y = 14.17x − 2.58	0.9992	0.59	1.54	1.07	2.41
Catechin gallate	280	36.00	3.00–48.00	y = 8.99x − 4.97	0.9993	1.14	2.49	1.41	1.36
Piceatannol	324	36.65	3.00–48.00	y = 39.74x − 0.73	1.0000	0.07	0.19	0.62	1.84
Viniferin	324	49.49	2.00–32.00	y = 27.64x − 14.29	0.9986	0.53	0.57	0.46	0.45

y: HPLC-DAD peak area of the corresponding to the compound; x: concentration of the corresponding compound (mg L^−1^); LOD: limit of detection; LOQ: limit of quantification; RSD: relative standard deviation.

**Table 2:** Oenological parameters of red grape samples at harvest under varying irrigation regimes.

Varieties	Irrigation Regime	Potential Alcohol Degree (% *v*/*v*)	pH	Total Acidity (g L^−1^) *
Tinta Gorda	C	12.70	3.79	4.58
	D	13.00	3.72	4.07
	R	12.40	3.77	3.94
Tinta Miúda	C	14.60	3.39	6.70
	D	15.80	3.36	6.75
	R	17.30	3.42	7.65
Tinta Caiada	C	11.60	3.62	5.32
	D	12.30	3.75	4.69
	R	13.70	3.91	4.45
Moreto	C	10.60	3.70	4.38
	D	8.40	3.71	4.16
	R	9.50	3.74	3.99

* Tartaric acid; C: water comfort; D: moderate water deficit; R: rainfed.

**Table 3:** Phenolic compounds (μg g^−1^) for the studied varieties at harvest under three irrigation regimes.

Compounds	Tinta Gorda			F-Ratio	Tinta Miúda			F-Ratio	Tinta Caiada			F-Ratio	Moreto			F-Ratio
	C	D	R		C	D	R		C	D	R		C	D	R	
**Anthocyanins**																
Delphinidin-3-glucoside	25.33 ± 1.75 a	26.45 ± 0.58 a	23.99 ± 0.66 a	3.55	34.11 ± 1.65 a	41.61 ± 0.86 c	37.09 ± 0.61 b	33.55	34.04 ± 1.06 a	36.27 ± 1.08 a	55.95 ± 1.30 b	329.85	12.13 ± 0.25 b	9.35 ± 0.63 a	11.55 ± 0.68 b	21.11
Cyanidin-3-glucoside	7.64 ± 0.62 a	7.62 ± 0.13 a	7.28 ± 0.32 a	0.72	8.83 ± 0.31 a	9.94 ± 1.00 a	8.75 ± 0.24 a	3.46	10.51 ± 0.06 b	9.23 ± 0.31 a	13.27 ± 0.68 c	68.88	6.45 ± 0.24 b	5.49 ± 0.14 a	5.92 ± 0.39 ab	8.95
Petunidin-3-glucoside	27.69 ± 2.11 a	30.9 ± 0.09 b	28.66 ± 0.37 ab	5.30	42.32 ± 1.57 a	48.06 ± 1.57 b	46.96 ± 0.49 b	16.13	54.27 ± 2.21 a	55.99 ± 1.17 a	81.97 ± 1.75 b	232.05	17.50 ± 0.57 c	12.86 ± 0.48 a	15.51 ± 0.16 b	83.92
Peonidin-3-glucoside	23.75 ± 2.31 a	28.09 ± 0.82 b	27.75 ± 0.61 b	8.20	83.4 ± 3.99 a	92.51 ± 2.78 b	88.21 ± 1.10 ab	7.52	12.65 ± 0.65 b	11.47 ± 0.47 a	17.07 ± 0.16 c	116.69	27.22 ± 0.35 c	17.77 ± 0.26 a	22.38 ± 0.31 b	722.88
Malvidin-3-glucoside	143.55 ± 11.48 a	161.93 ± 1.49 b	162.73 ± 3.84 b	7.13	456.16 ± 14.67 a	511.97 ± 6.51 b	561.72 ± 2.05 c	95.90	134.96 ± 4.49 a	143.15 ± 1.30 b	224.97 ± 1.91 c	874.22	152.52 ± 1.01 c	103.93 ± 1.27 a	141.19 ± 0.56 b	1984.39
Delphinidin-3-coumaroyl-glucoside	36.70 ± 2.80 a	44.67 ± 0.94 b	47.17 ± 0.83 b	28.65	79.11 ± 2.53 a	89.04 ± 0.66 b	100.64 ± 1.22 c	125.36	72.03 ± 1.86 a	82.75 ± 1.44 b	85.20 ± 2.04 b	45.51	23.89 ± 1.22 b	18.45 ± 0.75 a	24.28 ± 0.64 b	38.68
Anthocyanin derivate ^a^	20.43 ± 1.63 a	28.62 ± 2.96 b	30.11 ± 0.45 b	20.97	14.89 ± 0.05 a	16.77 ± 0.87 b	18.08 ± 0.46 c	23.62	33.14 ± 1.07 a	35.42 ± 0.19 b	35.54 ± 0.31 b	12.87	10.05 ± 0.56 b	7.74 ± 0.23 a	10.11 ± 0.52 b	25.92
Petunidin-3-coumaroyl-glucoside	27.71 ± 2.11 a	32.93 ± 1.72 b	31.59 ± 0.47 b	8.65	17.45 ± 0.41 a	19.81 ± 0.85 b	18.52 ± 0.24 a	13.21	70.32 ± 1.86 b	75.27 ± 1.97 c	66.69 ± 0.66 a	21.47	10.11 ± 0.35 c	8.81 ± 0.09 a	9.60 ± 0.10 b	27.80
Malvidin-3-coumaroyl-glucoside	154.12 ± 11.91 a	178.62 ± 4.23 b	178.77 ± 1.62 b	11.15	224.48 ± 4.44 a	229.63 ± 1.99 a	247.16 ± 1.15 b	50.83	166.38 ± 6.94 ab	173.37 ± 3.39 b	163.6 ± 1.17 a	3.73	87.98 ± 1.47 c	63.82 ± 1.46 a	78.69 ± 0.59 b	287.72
Total	466.93 ± 36.72 a	539.82 ± 12.96 b	538.05 ± 9.16 b	12.82	960.75 ± 29.62 a	1059.34 ± 17.11 b	1127.13 ± 7.56 c	96.37	588.31 ± 20.20 a	622.92 ± 11.33 b	744.27 ± 9.97 c	108.87	347.85 ± 6.02 c	248.22 ± 5.31 a	319.23 ± 3.95 b	778.71
**Flavanols**																
(+)-Catechin	23.01 ± 0.84 c	14.76 ± 0.49 b	11.37 ± 0.63 a	239.39	81.42 ± 1.62 b	72.71 ± 5.55 a	69.68 ± 1.19 a	9.58	45.74 ± 0.56 a	42.40 ± 3.83 a	43.00 ± 0.07 a	1.90	56.16 ± 0.81 b	60.66 ± 0.49 c	40.63 ± 0.49 a	880.23
Procyanidin derivative 1 ^b^	26.92 ± 4.26 a	25.56 ± 1.47 a	27.51 ± 0.60 a	0.43	56.93 ± 6.67 a	60.80 ± 7.04 a	67.38 ± 5.17 a	2.08	53.49 ± 1.12 c	47.38 ± 1.48 a	50.02 ± 1.12 b	17.99	62.56 ± 1.55 c	58.68 ± 0.59 b	40.75 ± 0.69 a	377.01
Flavanol 1 ^c^	82.91 ± 13.52 a	99.59 ± 7.63 a	120.17 ± 3.15 b	12.49	111.07 ± 1.61 a	116.23 ± 6.30 a	147.38 ± 5.59 b	46.65	88.57 ± 1.81 a	97.24 ± 2.59 b	108.58 ± 0.34 c	89.58	80.78 ± 1.98 a	92.90 ± 0.62 c	89.15 ± 0.51 b	75.90
(−)-Epicatechin	11.44 ± 3.28 a	8.71 ± 3.85 a	12.99 ± 1.49 a	1.53	16.13 ± 1.18 b	11.03 ± 0.86 a	18.89 ± 0.63 c	56.47	1.60 ± 0.12 b	1.93 ± 0.05 c	0.87 ± 0.12 a	86.40	13.04 ± 4.59 a	13.05 ± 1.00 a	11.17 ± 0.31 a	0.47
Procyanidin derivative 2 ^b^	83.76 ± 3.70 a	101.81 ± 0.77 b	117.89 ± 1.68 c	153.06	168.02 ± 1.50 a	190.49 ± 2.89 b	215.69 ± 1.56 c	392.85	89.55 ± 1.60 b	85.59 ± 0.12 a	98.56 ± 0.91 c	117.06	131.10 ± s37.29 b	109.71 ± 0.39 ab	87.28 ± 0.44 a	3.11
Epicatechin gallate	78.54 ± 2.82 ab	76.16 ± 0.44 a	80.23 ± 1.37 b	3.75	145.09 ± 1.30 a	154.58 ± 0.51 b	164.92 ± 3.72 c	56.14	112.56 ± 1.04 b	86.21 ± 1.19 a	87.40 ± 0.28 a	768.36	125.66 ± 11.41 b	113.22 ± 2.71 b	82.10 ± 1.12 a	32.64
Total	306.59 ± 28.43 a	362.60 ± 14.65 ab	370.16 ± 8.92 b	3.89	578.66 ± 13.88 a	605.84 ± 23.14 a	683.74 ± 17.86 b	15.05	391.51 ± 6.25 b	360.74 ± 9.27 a	388.43 ± 2.85 a	23.74	439.30 ± 57.63 b	448.22 ± 5.80 b	351.07 ± 3.56 a	49.97
**Flavonols**																
Flavonol 1 ^d^	34.31 ± 1.82 b	29.16 ± 1.01 a	32.38 ± 0.95 b	11.69	74.03 ± 2.85 a	91.70 ± 2.03 c	82.41 ± 0.89 b	53.92	57.35 ± 0.97 b	48.07 ± 0.37 a	61.94 ± 1.13 c	190.60	25.73 ± 0.78 a	31.33 ± 0.27 b	26.50 ± 0.25 a	111.42
Flavonol 2 ^d^	5.00 ± 0.23 a	5.58 ± 0.10 b	6.55 ± 0.29 c	37.78	7.75 ± 0.28 a	9.30 ± 0.09 b	9.41 ± 0.33 b	40.26	3.49 ± 0.04 a	4.19 ± 0.05 b	5.37 ± 0.17 c	243.27	2.54 ± 0.09 b	2.08 ± 0.10 a	2.49 ± 0.06 b	26.70
Total	39.31 ± 2.05 b	34.74 ± 1.11 a	38.93 ± 1.24 b	8.73	81.78 ± 3.13 a	101.01 ± 2.12 c	91.81 ± 1.22 b	53.69	60.84 ± 1.01 b	52.26 ± 0.42 a	67.31 ± 1.30 c	182.61	28.27 ± 0.87 a	33.41 ± 0.36 b	28.98 ± 0.32 a	80.48
**Others**																
Caffeic acid	1.38 ± 0.07 a	1.56 ± 0.06 b	1.50 ± 0.11 ab	3.73	2.36 ± 0.11 a	2.73 ± 0.11 b	2.91 ± 0.23 b	9.46	1.41 ± 0.02 a	1.48 ± 0.03 b	1.51 ± 0.04 b	8.60	2.30 ± 0.05 c	2.08 ± 0.16 b	1.88 ± 0.01 a	14.42
Resveratrol piceid	nd	nd	nd	--	3.06 ± 0.20 a	3.48 ± 0.60 a	3.41 ± 0.25 a	0.99	nd	nd	nd	--	nd	nd	nd	--
Total	1.38 ± 0.07 a	1.56 ± 0.06 b	1.50 ± 0.11 ab	3.73	5.42 ± 0.31 a	6.21 ± 0.71 a	6.32 ± 0.48 a	3.24	1.41 ± 0.02 a	1.48 ± 0.03 b	1.51 ± 0.04 b	8.60	2.30 ± 0.05 c	2.08 ± 0.16 b	1.88 ± 0.01 a	14.42

The concentration of phenolic compounds is expressed as mean ± SD; one-way analysis of variance (ANOVA) for each compound is included. Different letters within each column represent statistically significant differences among different irrigation regimes for each variety according to Tukey–Kramer multiple-comparison test (*p* < 0.05); C: water comfort; D: moderate water deficit; R: rainfed; ^a^ quantified relative to malvidin-3-glucoside; ^b^ quantified relative to procyanidin; ^c^ quantified relative to (+)-catechin; ^d^ quantified relative to quercetin; nd: not detected.

**Table 4:** Weights of the variables in the first two principal components for the principal component analysis (PCA) carried out with low-molecular-weight phenolic compounds for each irrigation regime.

Compounds	Water Comfort		Moderate Water Deficit		Rainfed	
	Component 1	Component 2	Component 1	Component 2	Component 1	Component 2
(+)-Catechin	0.2808	−0.0923	0.1871	−0.2102	0.2326	0.0238
Procyanidin derivative 1	0.1338	−0.1015	0.1469	−0.2075	0.2573	−0.0372
Flavanol 1	0.3130	0.0577	0.2935	0.0212	0.2673	−0.0053
Caffeic acid	0.1974	−0.2748	0.2445	−0.2308	0.2585	0.1806
Delphinidin-3-glucoside	0.1704	0.3147	0.2217	0.2677	0.1240	−0.3720
(−)-Epicatechin	0.1519	−0.2982	0.0499	−0.3674	0.1568	0.3258
Procyanidin derivative 2	0.2706	−0.1890	0.2802	−0.1360	0.2850	0.0878
Cyanidin-3-glucoside	0.0914	0.3577	0.2253	0.2613	0.0702	−0.3933
Petunidin-3-glucoside	0.1231	0.3442	0.1603	0.3192	0.0979	−0.3824
Peonidin-3-glucoside	0.2997	−0.0987	0.2882	−0.0719	0.2784	0.1391
Malvidin-3-glucoside	0.3118	−0.0455	0.2988	−0.0201	0.2950	0.0325
Resveratrol piceid	0.3132	−0.0281	0.2977	−0.0608	0.2861	0.1060
Epicatechin gallate	0.2513	−0.0955	0.2529	−0.2007	0.2896	0.0812
Flavonol 1	0.2706	0.2005	0.2973	0.0173	0.2737	−0.1463
Flavonol 2	0.2633	0.0415	0.2737	0.0861	0.2608	−0.0427
Delphinidin-3-coumaroyl-glucoside	0.2314	0.2596	0.2200	0.2569	0.2539	−0.2121
Anthocyanin derivate	−0.0740	0.3688	−0.0433	0.3899	−0.0201	−0.3627
Petunidin-3-coumaroyl-glucoside	−0.0752	0.3559	−0.0518	0.3630	−0.0273	−0.4087
Malvidin-3-coumaroylated-glucoside	0.2489	0.1963	0.2230	0.2382	0.2586	−0.0843

## Data Availability

The data presented in this study are available on request from the corresponding author.
